# 

**Published:** 1864-11

**Authors:** 


### REMOVAL.

☞ The Editorial and Private Office of DRS. MILLER and INGALS will hereafter be at No. 190 South Clark St., between Monroe and Adams Sts.

☞ Business letters to the Journal should be addressed to Drs. Miller & Ingals, Chicago, Ills., P. O. Drawer 5787. When money is received a receipt will be returned with the next number of the Journal, and subscribers failing to get such receipt will confer a favor by giving us notice of any omission

